# Correlation between gout and dry eye disease

**DOI:** 10.1007/s10792-024-02965-6

**Published:** 2024-02-20

**Authors:** Tongtong Chen, Jiaqi Chen, Cong Zhao, Xiang Li

**Affiliations:** 1 Eye School of Chengdu University of Traditional Chinese Medicine, Key Laboratory of Sichuan Province Ophthalmopathy Prevention and Cure and Visual Function Protection With Traditional Chinese Medicine Laboratory, Chengdu, 610075 Sichuan China; 2 Hospital of Chengdu University of Traditional Chinese Medicine, Chengdu, 610072 Sichuan Province China

**Keywords:** Dry eye disease, Gout, Inflammation, Oxidative stress

## Abstract

**Background:**

Gout is a common form of inflammatory arthritis that can cause a number of serious complications. Complications are common in patients with gout and complicate their management and disease outcome. The recent literature has reported that an increasing number of gout patients are presenting with dry eye symptoms. However, until now, the link between gout and dry eye disease has not been clearly defined. (It is unclear whether the two conditions simply co-exist, whether there are common risk factors, or whether dry eye disease is a complication of gout.)

**Methods:**

A thorough literature search was conducted in the PubMed database to summarize the most recent information on the correlation between gout and dry eye disease and to explore the potential relationship between the pathogenesis of the two. (*Objective*: Therefore, in this paper, we review the recent literature on the correlation between gout and dry eye disease and explore the potential association between the pathogenesis of both.)

**Results:**

Studies in the last five years have shown a correlation between gout and dry eye, i.e., gout is associated with an increased risk of dry eye. The NLRP3-IL-1β signaling pathway may be a potential mechanism for the combination of gout and dry eye disease; factors such as high blood uric acid and xanthine oxidase activation in gout patients may aggravate the development of dry eye disease; reducing the use of visual display terminals; reducing or abstaining from alcohol consumption; and moderate coffee intake may effectively prevent gout and dry eye disease.

**Conclusions:**

It is an undisputed fact that many gout patients present with dry eye manifestations that seriously affect the quality of life of gout patients, and early detection and treatment of dry eye in gout patients are crucial.

## Introduction

Gouty arthritis is the most common type of inflammatory arthritis in adults, and its incidence and prevalence have been increasing in recent years [1]. Gout is an inflammatory arthritis caused by the deposition of monosodium urate crystals in joints and non-joint structures and is characterized by hyperuricemia, urate crystal deposition, and inflammation [2]. The main risk factors for gout include hyperuricemia, genetics, comorbidities, medications, dietary factors, and lead exposure, with hyperuricemia being the most important risk factor for the development of gout [3]. Erythema, swelling and heat in joints and soft tissues, and even joint deformity are the main clinical manifestations of gout. In addition, the most common ocular symptom in gout patients is red eyes. Previous studies have confirmed that gout can cause ocular surface abnormalities, such as gouty stone deposits, subconjunctival hemorrhage, and vascular changes [4]. Ocular complications are caused by urate crystal deposits in ocular structures such as the eyelids [5], conjunctiva [6], aqueous humor, and iris [7] in gout patients. These affect the vision and quality of life of gout patients to varying degrees [8]. The association between gout and ocular diseases such as conjunctival congestion, uveitis [9], keratitis, glaucoma, and cataract [10] has also been reported. In recent years, there has been a gradual increase in the number of gout patients presenting with dry eye disease, and the correlation between gout and dry eye disease has raised concerns.

Dry eye disease (DED) is one of the most common ocular disorders affecting most of the population, with an impact range of 20–50%. DED is a heterogeneous disease of the ocular surface in which tear film instability, hyperosmolarity, neurosensory abnormalities, and ocular surface inflammation and injury are key etiologies, and these factors interact to form a vicious cycle leading to the chronic progressive development of the disease [11].

In this review, we assess the current state of the literature regarding the relationship between gout and DED in light of the available evidence, explore potential associations between gout and DED pathogenesis in terms of both inflammatory mechanisms and oxidative stress, and identify modifiable common risk factors for gout and DED.

(To this end, a series of literature searches were conducted in the PubMed database. The search strategy included a combination of medical subject headings and keywords. Search terms included “dry eye disease,” “dry eye,” “gout,” and “gouty arthritis,” and the study covered the period 1964–2023.)

## Correlation of gout with DED

Whether gout is a risk factor for DED and the association between the two has been a subject of much debate, and several studies have noted a strong association between DED and gout. In a Blue Mountains Eye Study published in 2003, which described the association between the prevalence of DED and systemic factors in an older Australian population (mean age 60.8 years, *n* = 1174), it was found that after adjusting for age and sex, gout was significantly associated with DED, and the time series and disease interval of gout may also show an effect on the development of DED [12].

In a study based on the Beaver Dam Eye Study cohort (*N* = 1993) published by Moss S E and colleagues in 2000, it was noted that a history of gout (OR 1.42; 95% CI 1.02–1.96) was independently and significantly associated with DED in a logistic model after controlling for age and sex [13]. However, examination of the prevalence of DED in the same cohort at five years revealed that the overall five-year prevalence of DED was not significantly associated with gout [14]. Assessing the 10-year incidence of DED in older adults, also based on this cohort at 10 years, noted no significant correlation between gout and DED [15]. This study was a longitudinal incidence study, which has the advantage over cross-sectional studies in that risk factors were observed prior to the onset of DED. This author hypothesized that dry eye may occur sequentially with gout, and that gout was associated with the incidence of DED. However, in a follow-up study, it was found that the association of DED incidence with gout failed to reach statistical significance after controlling for other factors. The explanation for the absence of a relationship between gout and DED incidence is that the study was unable to distinguish between tear deficiency and evaporative dry eye disease and did not subject participants to objective tests related to DED, instead of using a self-reported approach that lacked sensitivity and specificity to determine the presence of DED. In addition, the five-year and 10-year intervals are too long to possibly attempt to link gout to DED and to detect the incidence of resolved DED. In addition, the cohort represents an elderly population that has experienced attrition due to death both before and after the onset of DED. If both risk factors and DED are associated with mortality, then the results may be biased. Finally, because the cohort is a white middle-class population, the results may not be applicable to other populations of different races, socioeconomic classes, or geographic locations.

In a study published in 2017 using a multistage proportional sampling technique, gout was noted as one of the non-significant variables for DED [16]. In a retrospective population-based cohort study published in 2019, which analyzed data from 30,192 gout patients and 30,192 non-gout patients, a significantly increased risk of DED was observed in patients with gout (*p* = 0.001), and the study also considered the exposure period and found that gout increased the risk of DED after adjusting for other risk factors and that the risk was positively associated with a longer disease duration [17].

In a cross-sectional study published in 2021 by Selman Belviranli and colleagues that included 34 gout patients and 32 age- and sex-matched healthy individuals, patients with gout were found to be at greater risk for DED with ocular surface changes and tear film rupture abnormalities more commonly than non-gout patients [18]. Although the study had a relatively small sample size and a cross-sectional study design, it used the more reliable OSDI questionnaire for detecting DED and assessing its severity to assess the subjective symptoms associated with DED, and an objective test to assess the Schirmer 1 test, tear break-up time (TBUT), ocular surface disease index (OSDI) score, and CIC classification comparing the two groups.

In a study published in 2022 that used a Meta-analysis approach (630 studies involving 1.82 subjects were included) to identify potential risk factors for DED, gout (OR 0.001; *p* < 1.53) was found to be associated with an increased risk of DED, without significant heterogeneity for gout. It was also found that gout was not associated with the risk of DED if pooled studies were conducted in Eastern countries [19]. The disease distribution of gout differs between Eastern and Western countries, and environmental, dietary, and lifestyle factors in each country may influence the progression of DED. In addition, the threshold for age and the definitions of systemic disease, ophthalmic surgery, and DED differed among the studies included in the study, which could give rise to a potential uncontrolled bias. A retrospective case–control study published in the same year, which included a total of 973 and 1946 patients in the persistent gout and non-gout groups, found a significantly higher rate of DED in the persistent gout population (*p* = 0.0415) and suggested that a duration of gout longer than 10 years may be a threshold for a higher rate of DED presence [20].

The number of studies investigating the association between gout and DED is limited, the findings are contradictory, and it is not yet possible to clarify the correlation between gout and DED. However, the findings in the last five years are more biased toward the existence of some correlation between the two, i.e., gout is associated with an increased risk of DED. Most of the above observational studies on gout and DED are cross-sectional or prevalence studies, and they can only prove whether gout is associated with the incidence of DED but not the sequence of gout and DED onset and whether there is a causal relationship between them. Future large-scale studies are still needed to determine and evaluate the correlation and mechanism of association between gout and DED.

## Inflammatory mechanism

### The NLRP 3-IL-1 β signaling pathway

In recent years, many researchers have studied the pathogenesis of gout not in terms of purely metabolic diseases but in terms of inflammatory factors and immunology. Hyperuricemia caused by increased formation or decreased excretion of blood uric acid (SUA) is the main causative factor of gout, and when SUA levels exceed its saturation point, deposits of monosodium urate (MSU) crystals occur [21], which triggers the release of pro-inflammatory cytokines [22], triggering inflammatory responses such as vasodilation, leukocyte coagulation, and the production and release of tumor necrosis factor-α (TNF-α), interleukin-1β (IL-1β), interleukin 6 (IL-6), interleukin 8 (IL-8) [23], and interleukin 18 (IL-18) [24], leading to disorders in the expression of pro-inflammatory and anti-inflammatory factors, while inflammatory factors further cause infiltration of inflammatory cells and produce an inflammatory cascade response, thus inducing an acute attack of gouty arthritis [25]. Activation of NLRP3 inflammasome by uric acid monosodium salt crystals and release of IL-1β play a major role in the initiation of gout attacks [26]. Previous studies have shown that NLRP3 inflammatory vesicles activated by MSU crystals trigger inflammatory forms of cell death and trigger the release of the pro-inflammatory cytokine IL-1β [27], which in turn induces neutrophil infiltration of joints, leading to gout attacks with joint swelling and pain [28].

There is growing evidence that innate immune responses to various environmental stresses arise from ROS-induced activation of cytoplasmic NLRP3 inflammatory vesicles. DED is considered to be an inflammatory-mediated disease. Immune factors such as ocular surface inflammatory cells and inflammatory mediators play an important role in the pathogenesis of DED [29]. Factors such as hyperosmolarity, desiccation, and oxidative stress can activate ocular surface cell signaling pathways leading to the synthesis and release of pro-inflammatory cytokines (IL-1α [30], TNF-α, IL-1β, IL-18, and IL-6 [31]), tumor necrosis factor (TNF-α [32]), and matrix metalloproteinases (mainly MMP9) [33]. A study proposed that the ROS-NLRP3-IL-1β signaling pathway may play an initiating role in the environmentally induced progression of dry eye disease, with elevated ROS production triggering NLRP3 inflammatory vesicle complex and activation, leading to increased secretion of bioactive IL-1β, which in turn induces the development of DED [34].

In a pilot study of patients with refractory gouty arthritis, the IL-1 receptor antagonist anakinra was found to provide rapid relief of gouty inflammatory symptoms [35]. When conventional treatment is not appropriate, anakinra is an effective option for patients with gout attacks [36]. In addition, data from a prospective, double-blind, randomized trial showed that topical application of anakinra was effective in significantly reducing symptoms and corneal epithelial lesions in patients with DED-related conditions [37]. Therefore, IL-1 receptor antagonists may be a common target for gout and dry eye and may be a novel therapeutic option for patients with gout combined with DED.

**Fig. 1:**
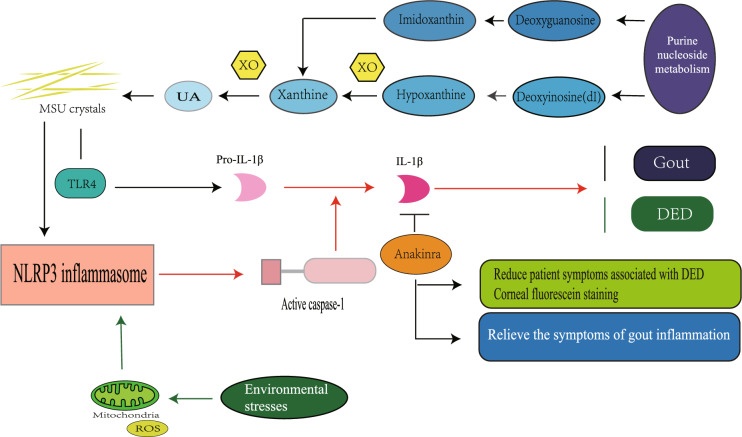
The NLRP 3-IL-1 β signaling pathway in the pathogenesis of gout and DED

It was found that tear uric acid levels were higher in gout patients than in non-gout patients, and tear uric acid values were significantly and positively correlated with tear IL-1β levels, suggesting an interaction between gout and ocular inflammatory response [38]. Ocular inflammation is a rare but well-established consequence of gout and also plays an important role in the pathophysiology of DED. The types of immune responses, pro-inflammatory cytokines, inflammatory factors, and disease processes involved in gout and dry eye are very similar, and there may be common or similar inflammatory pathways in the pathogenesis of both. The NLRP3-IL-1β signaling pathway may play a role in the progression of gout combined with DED, and aberrant activation of NLRP3 inflammatory vesicles may be an initiator of gout and DED, and strategies that affect its activity or impede its activation could effectively inhibit the associated inflammatory response. Topical application of NLRP3 inflammasome inhibitors and IL-1 receptor antagonists may be a new modality in the current treatment of gout combined with DED (Fig. 1).

### Matrix metalloproteinase 9 (MMP-9)

MMP-9 is induced by key cytokines in the early stages of the inflammatory cascade response and is an ideal biomarker [39], and its expression levels are closely related to the progression of gout and DED. MMP-9 expression is increased in both gout patients and dry eye patients. Gout patients stimulate the production of MMP-9 directly or indirectly by macrophages due to urate crystals in the body, which in turn causes inflammation and tissue damage. Increased expression of MMP-9 in gout patients may be involved in the degradation of ECM components in gouty arthritis [40], and its increased activity may lead to gout attacks [41]. The concentration and activity of MMP-9 were found to be significantly increased in the tears of patients with DED, and it plays an important role in the pathogenesis of DED, leading to corneal dysregulation, corneal barrier dysfunction, reduced tear production, and desquamation [42]. Tear hypertonicity triggers the release of MMP-9, which in turn triggers a progressive inflammatory cycle [43]. It has been suggested that positive detection of MMP-9 is associated with reduced long-term tear production [44] and that elevated concentrations of it in tears are directly related to the diagnosis of DED [45].

Some studies support that quercetin significantly reduces the relative expression of MMP-9 in gout patients [46] and has the potential to be developed as a dietary supplement for the prevention and management of gouty arthritis [47]. In addition, topical application of quercetin was found to significantly reduce MMP-9 levels and improve dry eye ocular surface disorders in a mouse model of dry eye [48]. MMP-9 may serve as a biomarker and risk predictor for gout and DED, and modulation and inhibition of MMP-9 may be an important therapeutic strategy for gout and DED.

## Oxidative stress

Cellular and tissue damage due to oxidative stress is one of the initiating factors in the development of many diseases, and the important role of oxidative stress in the pathogenesis of DED has been well established. Due to its long-term exposure, the ocular surface is the most vulnerable to attack by various physicochemical factors, and the common result of these attacks is an increase in intracellular reactive oxygen species, leading to oxidative stress in ocular surface tissues. Excessive ROS release significantly decreases cell viability, which promotes inflammation through a positive feedback loop in the DED, thereby increasing the symptoms of DED.

Xanthine oxidase is an important enzyme in purine metabolism and is a form of xanthine oxidoreductase [49]. The role of xanthine oxidase in the development of gout is well established, as it plays an important role in catalyzing the oxidative hydroxylation of hypoxanthine to xanthine and further catalyzing the production of uric acid (UA) from xanthine [50]. When xanthine oxidase activity is abnormally high, large amounts of uric acid are produced. When the concentration of uric acid in body fluids exceeds its saturation concentration, it tends to precipitate crystals in the tissues, resulting in hyperuricemia and gout. Uric acid is the end product of purine metabolism in humans and has both antioxidant and pro-oxidant effects in the body. At physiological concentrations, UA is a potent antioxidant that scavenges ROS and protects cells from oxidative stress. However, UA has a limited ability to scavenge free radicals, and under certain conditions, it disrupts the oxidation–reduction homeostasis of the body through a variety of pathways, resulting in an oxidative stress state in the body.

The production of ROS by increased activity of xanthine oxidase during uric acid metabolism is one of the mechanisms by which oxidative stress accumulates under conditions of hyperuricemia. During urate production, the catalytic enzyme xanthine oxidase produces large amounts of ROS that affects biological cellular mechanisms and regulates cellular redox status and cell signaling through the production of ROS, the dysregulation of which may lead to oxidative stress and induce cellular damage.

Several studies have found significant expression and activity of xanthine oxidoreductase/xanthine oxidase in the conjunctival epithelium and tear fluid of patients with DED [51–53]. Xanthine oxidase is a key source of reactive oxygen species, and when xanthine oxidase is present in large amounts in the conjunctival epithelium, the reactive oxygen products produced by these enzymes can promote oxidative reactions at the ocular surface and exacerbate dry eye symptoms. The factors that induce this enzyme system in DED are complex and include inflammatory mediators, mechanical stress, and hypoxia. Reduced tear production and dryness of the ocular surface in DED patients lead to mechanical irritation, and mechanical stress activates xanthine oxidoreductase through a mitogen-activated protein kinase-dependent pathway, which may promote ocular surface inflammation. Mechanical irritation also causes ocular surface cell damage and hypoxia in patients with dry eye, and moderate hypoxia in ocular surface cells also increases xanthine oxidoreductase immunoreactive protein levels and xanthine oxidoreductase activity [51–53]. A novel artificial drop based on a combination of arabinogalactan and hyaluronic acid was found to reduce or inhibit xanthine and xanthine oxidoreductase reactions, thereby significantly reducing uric acid and reactive oxygen species formation in vitro, which may contribute to the treatment of DED [54].

Whether or not gout is considered a risk factor for DED, we know that its formation pathway and its intracellular reactions lead to oxidative stress, which is thought to be an important factor in the progression of DED. Factors such as high blood uric acid and activation of xanthine oxidase expression in gout patients may be involved in or induce oxidative stress in ocular surface tissues, exacerbating the progression of DED and perhaps being a potential mechanism for gout combined with DED. Xanthine oxidase is an important cause of elevated uric acid values and gout formation, an important target for gout treatment, and also plays a role in the pathogenesis of DED. Inhibition of xanthine oxidase is the effective and most prominent treatment for gout [41] and is a safe and reliable therapeutic strategy for the treatment of gout combined with DED.

## Common risk factors for gout and DED

Gout and DED are both lifestyle-related diseases. When clinical treatment plans for gout and DED are developed, they should not be limited to inflammation control and uric acid-lowering therapy, but can be based on interventions in daily habits, diet, exercise, and sleep to improve lifestyle, quality of life, remission, and prognosis. Epidemiologically, gout and DED share many risk factors, including persistent alcohol consumption, advanced age, diabetes, hypertension, and dietary factors. The identification of modifiable risk factors is critical for effective treatment and prevention of gout and DED.

Moderate coffee intake may be advocated for DED and gout prevention. Coffee is one of the most widely consumed beverages in the world and may influence the risk of developing gout and DED through a variety of mechanisms. Coffee has been evaluated several times as a risk factor for DED in general prevalence and risk factor studies. Although the effect of caffeine, the main component of coffee, on lacrimal gland function and DED is uncertain, several studies have pointed out that caffeine use is one of the factors associated with a lower incidence of DED, that higher caffeine intake is associated with a slightly reduced risk of DED [55], and that increased caffeine intake is a protective factor for DED [56]. It has also been found that oral caffeine appears to stimulate tear production in healthy non-dry eye disease subjects [57].

A Meta-analysis showed that coffee had a significant reduction in serum uric acid and that moderate coffee intake may be advocated for primary prevention of gout [58]. Some prospective data suggest that long-term coffee consumption is associated with a lower risk of gout [59, 60] and that coffee consumption has a strong nonlinear association in reducing the risk of gout [61]. In addition, one study found that coffee consumption was associated with lower serum uric acid levels and frequency of hyperuricemia, and the negative association with coffee seems to be through coffee components other than caffeine [60]. The effects of coffee on gout patients are complex, and the role and mechanisms by which coffee can reduce the risk of gout remain to be investigated. One explanation suggests that caffeine, the main component of coffee, which is a naturally occurring trimethylxanthine alkaloid, belongs to the methylxanthine family and is a competitive inhibitor of xanthine oxidase. Coffee can reduce uric acid production by interfering with the oxidation of xanthine to produce urate. However, there was also a significant association between decaffeinated coffee and lower uric acid, suggesting that other components of coffee besides caffeine may also contribute to the reduction of uric acid.

Visual display terminal (VDT) users and continued alcohol consumption were found to be more associated with DED in the persistent gout population than in the non-gout population. VDT use and continued alcohol consumption were the main lifestyle and symptom risk factors for DED in patients with gout for more than 10 years [20]. Visual display terminals, continued alcohol consumption, and reduced caffeine consumption are modifiable lifestyle factors that put gout and DED at increased risk. Reduced VDT use, reduced alcohol consumption or abstinence, and moderate coffee intake are effective in preventing the development of gout and DED.

## Results and discussion

Gout is a chronic progressive inflammatory arthritis with a high rate of comorbidity and disability. In recent years, the prevalence of gout has continued to increase, and the increasing number of gout patients has placed a tremendous burden on society and families, making breakthroughs in gout diagnosis and treatment urgent. DED is one of the most common chronic multifactorial ocular surface diseases with a high prevalence and complex pathogenesis. DED causes ocular discomfort and potential damage to the ocular surface, severely reducing patients' life treatment and work efficiency. Although the current evidence does not fully support the association between gout and DED, it is indisputable that many gout patients present with dry eye manifestations, which seriously affects the quality of life of gout patients. It has become urgent to clarify the association between gout and dry eye and the related pathophysiology and to find effective treatment options.

This is the first review to explore the correlation between gout and dry eye and related mechanisms. This review firstly summarizes the progress of research on the correlation between gout and dry eye, and although the correlation is not conclusive, the possible association between the two cannot be ignored. Long-term and large-scale studies are still needed to evaluate whether dry eye is a common comorbidity of gout, whether gout is a risk factor for dry eye, and the pathophysiological mechanisms associated with both. Second, the molecular mechanisms associated with gout and dry eye were explored in terms of inflammatory mechanisms and oxidative stress, respectively, and it was found that similar inflammatory cytokines and inflammatory pathways seem to be involved and act through a complex network of factors to cause gout combined with dry eye, and inhibition of common or similar inflammatory pathways and inflammatory factors is a promising therapeutic target for gout combined with dry eye. Gout formation pathways and their intracellular responses leading to oxidative stress may be important factors in promoting the progression of dry eye. Then, reducing VDT use, alcohol consumption, and moderate coffee intake could be used for dry eye and gout prevention. Finally, early detection and treatment of dry eye in gout patients are crucial, and ophthalmic assessment can be used to improve early recognition and management of dry eye in gout patients, and regular eye examinations are recommended for long-term gout patients with a view to early detection of DED and prompt treatment.
